# Association Between Dietary Inflammatory Index and Depression Symptoms in Chronic Kidney Disease

**DOI:** 10.1155/bn/9253956

**Published:** 2025-03-07

**Authors:** Rui Huang, Qixia Zhu

**Affiliations:** ^1^College of Nursing, Weinan Vocational and Technical College, Weinan, Shaanxi, China; ^2^Department of Nursing, The First Affiliated Hospital of Air Force Military Medical University, Xi'an, Shaanxi, China

**Keywords:** chronic kidney disease, depression, dietary inflammatory index, NHANES

## Abstract

**Objective:** The study is aimed at investigating the relationship between dietary inflammatory index (DII) score and depression symptoms in chronic kidney disease (CKD), exploring its potential role as an indicator of depression risk and offering new insights into dietary interventions for this vulnerable population.

**Materials and Methods:** The cross-sectional investigation included CKD patients aged ≥ 18 in the 2007–2018 National Health and Nutrition Examination Survey. The Patient Health Questionnaire-9 (PHQ-9) was administered to evaluate depression symptoms. Dietary information was obtained from a 24-h dietary recall interview. The relationship between DII and depression was explored through weighted univariate and multivariate logistic regression models, adjusting for relevant covariates identified via backward selection. Results were expressed as odds ratios (ORs) with 95% confidence intervals (CIs). To further investigate the association, restricted cubic spline (RCS) and subgroup analyses were conducted.

**Results:** Totally, 489 (11.55%) patients with CKD had depression symptoms. A high DII score was linked to elevated depression symptoms incidence in CKD (OR: 1.67, 95% CI: 1.06–2.65). Adjusting all covariates, the relationship between DII score and depression symptoms still existed in patients aged ≥ 60 years (OR: 1.80, 95% CI: 1.16–2.79), males (OR: 2.05, 95% CI: 1.16–3.59), smokers (OR: 1.70, 95% CI: 1.06–2.75), and those without sleep disorders (OR: 1.81, 95% CI: 1.01–3.23).

**Conclusion:** DII score was associated with depression symptoms in patients with CKD. The findings suggest that diet plays a role in mental health, particularly in chronic conditions like CKD. The results underscore the importance of exploring anti-inflammatory dietary interventions to mitigate depression symptoms in this population. Further longitudinal research is necessary to establish causality and determine the efficacy of targeted dietary modifications in CKD patients with depression.

**Limitations:** As a cross-sectional study, causality cannot be inferred from these findings. Additionally, the reliance on self-reported dietary data may introduce bias, and unmeasured confounders could influence the observed associations.

## 1. Background

Chronic kidney disease (CKD) is a growing health issue affecting 10% of the global population, with patients experiencing both physical and mental challenges [1]. Depression has emerged as a particularly prevalent comorbidity among patients with CKD, affecting approximately 26.5% of them [2–5]. Depression is linked to various adverse outcomes, such as increased mortality, higher hospitalization rates, and reduced treatment adherence and quality of life [6]. The intricate relationship between inflammation, diet, and mental health has garnered attention recently. Yet, the nuances of these interactions remain inadequately explored, especially within the specific context of CKD. The elevated burden of depression in CKD is thought to be partially due to the chronic inflammation seen in these patients, which may contribute to altered neurotransmitter metabolism and neuronal function, thereby promoting depressive symptoms [5]. Nonetheless, the precise role of diet in modulating inflammation and its potential impact on depression in this population has been relatively unexplored.

The dietary inflammatory index (DII) was created to evaluate the proinflammatory or anti-inflammatory characteristics of an individual's diet, with higher scores indicating a more proinflammatory diet [7]. The association between high DII scores, indicative of proinflammatory diets, and depression symptoms has been reported across diverse populations. Elevated DII score was related to increased depression risk in the general population [8, 9]. In addition, higher DII scores were also associated with elevated odds of depression in several inflammatory diseases, including hypertension, diabetes, and coronary heart disease [10]. Yan et al. [11] also reported a link between higher DII scores and higher odds of all-cause mortality in CKD. However, previous studies do not consider the pathophysiological changes and dietary restrictions characteristic of kidney disease.

CKD patients exhibit distinct patterns of dietary intake that often increase their susceptibility to chronic inflammation. This heightened inflammatory state is attributable not only to the kidney's impaired ability to regulate the body's homeostasis but also to the dietary modifications necessitated by the disease. Common dietary recommendations for individuals with CKD often necessitate restrictions on potassium, phosphorus, and protein intake, which can inadvertently lead to suboptimal nutrient intake and worse inflammatory profiles [12, 13]. The lack of micronutrients coupled with the effects of dialysis of other treatments further complicates the dietary landscape for these patients, creating a unique context in which diet becomes a pivotal factor in the management of both inflammation and mental health symptoms. Therefore, exploring the association between DII and depression is not only pertinent but also imperative to tailoring effective interventions.

This study is aimed at bridging the gap in the existing literature by focusing explicitly on the associations between DII and depressive symptoms among individuals with CKD. By advancing our understanding of these relationships, this research is aimed at informing both dietary interventions and mental health strategies for improving the quality of life in individuals living with CKD.

## 2. Materials and Methods

### 2.1. Study Population

Data on patients with CKD were extracted based on the Nation Health and Nutrition Examination Survey (NHANES) for this cross-sectional study. The nationally representative survey NHANES collected information through interviews and physical examinations. Conducted by the National Center for Health Statistics (NCHS), the study assesses the health and nutritional status of adults and children across the United States. The NHANES protocol received approval from the NCHS Ethics Review Board. The study was exempt from review by the ethics committee at Weinan Vocational and Technical College, as the data was sourced from a public database.

Seven cycles of the NHANES (2007–2018) were used in the study. Patients with CKD were determined as those with an estimated glomerular filtration rate (eGFR) < 60 mL/min/1.73 m^2^ and/or a urinary albumin–Cr ratio (ACR) > 30 mg/g [14]. Individuals who met any of the following criteria were excluded: (1) without complete information of 24-h dietary recall, (2) without complete information of the Patient Health Questionnaire-9 (PHQ-9), and (3) missing information of body mass index (BMI).

### 2.2. Depression Symptom Assessment

Depressive symptoms over the past 2 weeks were assessed utilizing PHQ-9. Trained interviewers conducted evaluations via a computer-assisted system at a mobile center. The nine PHQ-9 items assessed symptom frequency: (1) anhedonia, (2) depressed mood, (3) sleep disturbance, (4) fatigue, (5) appetite changes, (6) low self-esteem, (7) concentration issues, (8) psychomotor disturbances, and (9) suicidal ideation. Total scores ranged from 0 to 27, with 10 or more scores indicating clinically significant depressive symptoms [15].

### 2.3. DII Assessment

DII is a comprehensive scoring system created to assess the potential inflammatory impact of dietary intake. According to the previous studies [16, 17], the DII score was derived from 27 food parameters obtained from NHANES 2007–2018. Parameters used for calculation include energy; carbohydrate; protein; total fat; dietary fiber; cholesterol; saturated, monounsaturated, and polyunsaturated fatty acids; *ω*-3 and *ω*-6 polyunsaturated fatty acids; vitamins A, B_1_, B_2_, B_3_, B_6_, B_12_, C, D, and E; folic acid; alcohol; *β*-carotene; caffeine; iron; magnesium; zinc; and selenium. The mean and variability of each nutrient were sourced from the World Diet Standards Library and utilized to transform the *Z*-scores of the respective nutrients into *Z*-transformed scores [18]. Each nutrient's *Z*-transformed value was then converted into percentiles to minimize the influence of outliers and right skewing, with the final distribution for each nutrient adjusted to be symmetrical around 0 by doubling the transformed percentiles and subtracting 1. The distribution limits varied from −1 (indicating maximum anti-inflammatory) to +1 (indicating maximum proinflammatory). Finally, the DII score is derived by multiplying the level of each nutrient level by its respective inflammatory fraction and adding the outcomes [18]. Higher positive scores indicate a more proinflammatory diet, while lower negative values correspond to a stronger anti-inflammatory impact [19].

### 2.4. Covariates

Covariates included age, gender, race, smoking, sleep disorders, cardiovascular disease (CVD), and psychotherapeutic agents. Race was categorized as non-Hispanic White, non-Hispanic Black, or other. Smoking status was defined as having smoked over 100 cigarettes, self-reported during the household interview [20]. Sleep disorders were identified if participants confirmed having informed a healthcare professional about sleep difficulties [21]. CVD was self-reported based on diagnoses or cardiovascular medication use. Participants were considered CVD-positive if they answered “yes” to questions about heart conditions, such as angina, heart attack, coronary heart disease, stroke, or congestive heart failure [22]. Psychotherapeutic agent use was determined from the questionnaire “Do you take medication for depression?” and data extracted on prescription medication use from the database [23].

### 2.5. Statistical Analysis

The sampling weights SDMVPSU, SDMVSTRA, and WTDRD1 were applied. Means and standard errors (SE) were reported for continuous variables, while counts and percentages were presented for categorical variables. Weighted *t*-tests and chi-square tests were employed to analyze continuous and categorical variables, respectively, to examine between-group differences in depression symptoms. Weighted univariate and multivariate logistic regression models were used to assess the association between DII score and depression symptoms among CKD patients, with results expressed as odds ratios (ORs) and 95% confidence intervals (CIs). Covariates were selected using the backward selection method in the weighted univariate logistic regression model. Model 1 was the crude model with no adjustments. Model 2 adjusted for age, gender, and race. Model 3 adjusted for all potential covariates, including age, gender, race, smoking, sleep disorders, CVD, and psychotherapeutic agents (Table S1). Restricted cubic spline (RCS) analysis was performed to examine the relationship between DII scores and depression symptoms in CKD patients.

Additionally, subgroup analyses stratified by age, gender, smoking, and sleep disorders were conducted to assess the stability of the relationship between DII score and depression symptoms. Missing variable values are shown in Table S2, with missing data addressed using the random forest chain equation multiple imputation method. No significant differences were observed in the data before and after imputation, indicating that imputation did not influence variable distribution (Table S3). All analyses were performed using SAS 9.4 (SAS Institute Inc., Cary, North Carolina, United States) and Python 3.9, with a *p* value < 0.05 considered statistically significant.

## 3. Results

### 3.1. Characteristics of Patients With CKD

**Figure 1:**
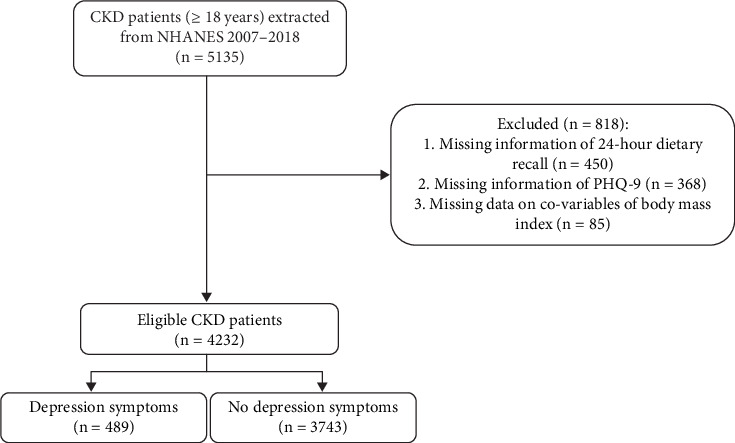
The flow chart of included chronic kidney disease patients.

**Figure 2:**
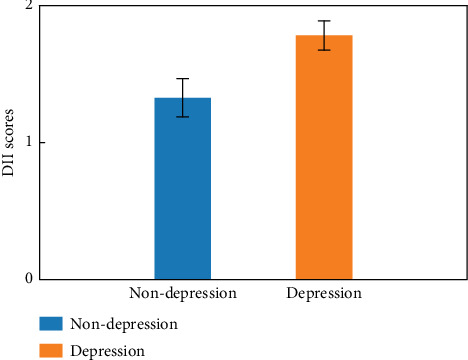
The mean DII score in patients with and without depression.

The characteristics of CKD patients are presented in Table 1, while the screening process is depicted in Figure 1. A total of 5135 CKD patients were included in the dataset. Of these, 903 participants were excluded due to incomplete information, including 450 with missing 24-h dietary recall data, 368 with missing PHQ-9 scores, and 85 with missing BMI data. Consequently, 4232 CKD patients were included in the final analysis, with a mean age of 57.80. The mean DII score was 1.38, with tertiles as follows: < 0.65, 0.65–2.41, and ≥ 2.14, respectively. Depression symptoms were present in 489 (11.55%) participants. The mean DII score was higher in patients with depression than those without depression (Figure 2). Those with depression were more likely to be female. Patients with depression symptoms exhibited a higher percentage of lower PIR, smoking, alcohol consumption, sleep disorders, diabetes, hypertension, dyslipidemia, and CVD. Statistical differences between the depression and nondepression groups were observed in DII score, age, gender, race, education, marriage, poverty income ratio, smoking, physical activity, sleep disorders, thyroid disease, diabetes, hypertension, dyslipidemia, CVD, BMI, white blood cell, albumin, dialysis, nephrotoxic drugs, and psychotherapeutic agents (all *p* < 0.05).

### 3.2. Association With DII Score of Depression Symptoms in CKD

**Figure 3:**
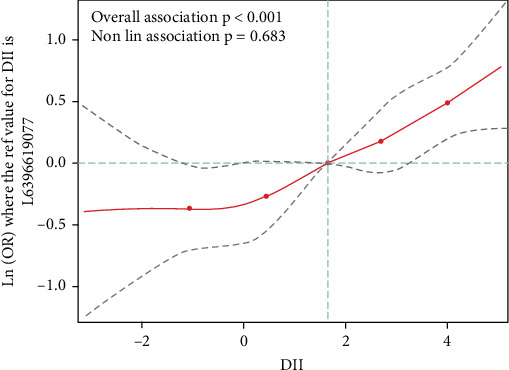
The relationship of DII score with depression symptoms in all chronic kidney disease patients.

As shown in Table 2, high DII score was linked to elevated odds of depression symptoms after being adjusted for age, gender, race, smoking, sleep disorders, CVD, and psychotherapeutic in Model 3 (OR: 1.67, 95% CI: 1.06–2.65). Moreover, elevated DII score was related to a higher incidence of depression symptoms (all *p* trend < 0.05). As depicted in Figure 3, the spline variable indicated that the DII score was not nonlinearly related to the likelihood of depression symptoms (*p*_nonlinear_ = 0.68).

### 3.3. Association of DII Score With Depression Symptoms Among Age, Gender, Smoking, and Sleep Disorder Subgroups

**Figure 4:**
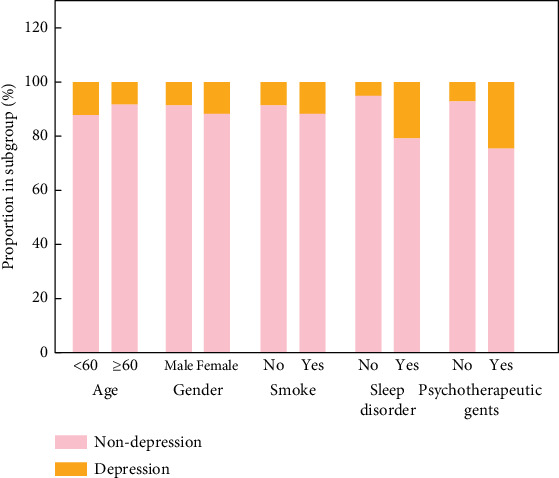
The proportion of patients with depression in different subgroups.

The relationships between DII score and depression symptoms in different subgroups are shown in Table 3. When DII was a continuous variable, a high DII score was related to a higher incidence of depression symptoms in male patients (OR: 2.05, 95% CI: 1.16–3.59) and those without sleep disorders (OR: 1.19, 95% CI: 1.01–1.42). Considering DII as a categorical variable, DII score ≥ 2.14 was related to a higher incidence of having depression symptoms in those age ≥ 60 years old (OR: 1.80, 95% CI: 1.16–2.79), males (OR: 2.05, 95% CI: 1.16–3.59), smokers (OR: 1.70, 95% CI: 1.06–2.75), and those without sleep disorders (OR: 1.81, 95% CI: 1.01–3.23). In addition, the proportion of patients with depression in subgroups is presented in Figure 4 and Table S4.

## 4. Discussion

Our findings indicate that a higher DII score, reflecting a proinflammatory diet, is associated with an increased incidence of depression symptoms in patients with CKD. The association remained robust across several subgroups, including patients aged ≥ 60 years, males, individuals with a smoking habit, and those without sleep disorders. These results suggest that dietary inflammation may play a significant role in the mental health of CKD patients, highlighting the importance of dietary management as a potential avenue for alleviating depressive symptoms in this vulnerable group.

The association between DII score and depression symptoms has been widely explored in previous studies [8, 9, 24, 25]. Luo et al. [9] reported that a proinflammatory diet is associated with a higher prevalence of depression in the US population, supporting our findings of a positive correlation between elevated DII score and depression symptoms. The study conducted among older adults in China found that a diet rich in anti-inflammatory foods was associated with a lower incidence of depressive symptoms, which aligns with our findings in the subgroup of individuals aged ≥ 60 years [26]. Additionally, Shakya et al. [27] suggested that a less inflammatory diet could improve depression symptoms, further corroborating the potential beneficial role of dietary interventions in managing depression. Zhang et al. [8] also reported a positive relationship between higher DII scores and depression symptoms, noting that both elevated DII and depression were linked to increased all-cause and CVD mortality risk in the general population. While the existing literature consistently supports the detrimental effects of a proinflammatory diet on mental health, our study contributes to this body of knowledge by demonstrating that this relationship persists across various subgroups, including individuals aged ≥ 60 years, males, smokers, and those without sleep disorders. Dietary interventions, particularly those aimed at reducing inflammation, appear to be crucial for the prevention and management of depression symptoms in patients with CKD [28, 29].

The stronger associations in males may reflect sex-related differences in inflammatory responses, with males exhibiting higher baseline levels of inflammation compared to females, which were different from a previous study [30]. This may be due to the limitations of our sample size in the subgroups. Smoking, known to exacerbate systemic inflammation, may further amplify the inflammatory burden in smokers, thereby intensifying the link between diet and depression symptoms. In the subgroup of individuals aged ≥ 60 years, the observed relationship could be influenced by age-related changes in immune function, where chronic low-grade inflammation becomes more prominent and may interact with dietary factors to worsen mood symptoms. Additionally, individuals without sleep disorders might have a more direct inflammatory response to diet, as sleep disturbances are often associated with increased inflammation and may confound the relationship between diet and depression. These subgroup differences highlight the need for targeted interventions that consider specific patient characteristics to better manage depression symptoms in the context of CKD.

The observed relationship between the DII score and depression symptoms in patients with CKD can be attributed to several potential mechanisms, particularly inflammation, oxidative stress, brain-derived neurotrophic factor (BDNF), and tryptophan/serotonin metabolism. Chronic inflammation, commonly present in CKD, is known to contribute to the development of depressive symptoms in this population [29, 31]. Proinflammatory diets, characterized by high intakes of processed foods, saturated fats, and refined sugars, promote inflammation and increase oxidative stress [32]. Inflammatory and oxidative processes can lead to neuronal damage, altering neurotransmitter systems involved in mood regulation, thus potentially contributing to depression in CKD patients [33]. Furthermore, the neurotrophic hypothesis suggests that compromised levels of BDNF, a key player in neuronal survival and plasticity, are linked to the pathophysiology of depression [34]. Among CKD, inflammatory and oxidative stress may downregulate BDNF production, influencing mood regulation [35]. Research has shown that dietary patterns enhancing BDNF levels may improve depression outcomes, suggesting a potential link between diet and mental health [36]. Finally, tryptophan, a precursor to serotonin, plays a crucial role in mood regulation [37]. Inflammatory states can reduce tryptophan availability and alter the activity of tryptophan-metabolizing enzymes, thereby impairing serotonin synthesis (a neurotransmitter implicated in mood regulation) [37]. A diet with a higher inflammatory potential, reflected by a higher DII score, may hinder tryptophan availability and subsequently contribute to depressive symptoms in CKD. More research is necessary to clarify the exact pathways and to explore targeted dietary interventions that could mitigate inflammation and improve mental health outcomes in CKD.

Due to the elevated prevalence of depression in CKD and its negative effects on overall health and quality of life, the identification of modifiable risk factors like diet is essential. Healthcare providers should consider incorporating dietary interventions aimed at reducing inflammation and improving mental health outcomes in patients with CKD. Specifically, nutritional counseling focused on the adoption of anti-inflammatory diets, such as the Mediterranean diet or plant-based eating patterns rich in fruits, vegetables, whole grains, and omega-3 fatty acids, may be beneficial. Additionally, reducing the intake of proinflammatory foods, including processed meats, refined carbohydrates, and excess salt, could further support mental well-being. Integrating these dietary strategies into a personalized care plan, along with regular monitoring of dietary adherence and mental health status, could play a crucial role in improving both physical and psychological outcomes for CKD patients, particularly those at risk for depression.

Several limitations must be acknowledged. Primarily, the cross-sectional design limits causality or temporal relationships between DII score and depression symptoms. Longitudinal and intervention studies are needed to confirm findings in patients with CKD. Additionally, the reliance on a single 24-h dietary recall interview may introduce recall bias, as it may not accurately reflect habitual dietary patterns. This method is susceptible to underreporting or overreporting of dietary intake, particularly in populations with chronic conditions such as CKD. Future studies should use more objective dietary measures, such as biomarkers or multiple-day dietary records, for better accuracy. In addition, the selection of the 27 food parameters used to calculate the DII is based on prior and expert consensus; however, the specific justification for the inclusion of these food items in the context of CKD and depression symptoms is not fully discussed. While DII is widely used to assess dietary inflammation, it does not account for all dietary components that influence inflammation or mental health. The limitations inherent in the DII as a dietary measure, such as the lack of consideration for portion sizes, cooking methods, and other dietary nuances, should be acknowledged. Unmeasured variables, such as socioeconomic status and medication adherence, may influence both dietary patterns and depression symptoms in CKD patients. Finally, the generalizability of our findings to other populations with chronic diseases remains uncertain, as the study sample is limited to individuals with CKD.

## 5. Conclusion

We found an association between higher DII scores and a higher incidence of depression symptoms among patients with CKD. The findings indicated the importance of diet in managing mental health outcomes for patients with CKD and suggested that anti-inflammation dietary interventions may be beneficial to improve mental health. Further studies are needed to explore the validation of dietary interventions and verify our findings.

## Figures and Tables

**Table 1:** Characteristics of CKD patients.

**Variables**	**Total (** **n** = 4232**)**	**Depression (** **n** = 489**)**	**No depression (** **n** = 3743**)**	**Statistics**	**p**
DII score, mean (SE)	1.38 (0.04)	1.79 (0.11)	1.33 (0.04)	*t* = −3.89	< 0.001
DII score, *n* (%)				*χ* ^2^ = 16.48	< 0.001
< 0.65	1239 (33)	101 (24)	1138 (34)		
0.65–2.14	1407 (34)	155 (31)	1252 (34)		
≥ 2.14	1586 (33)	233 (45)	1353 (32)		
Age (years), mean (SE)	57.80 (0.42)	56.39 (0.87)	57.96 (0.47)	*t* = 1.55	0.125
Age, *n* (%)				*χ* ^2^ = 7.90	0.005
< 60	1657 (47)	227 (56)	1430 (46)		
≥ 60	2575 (53)	262 (44)	2313 (54)		
Gender, *n* (%)				*χ* ^2^ = 5.41	0.020
Male	1985 (42)	184 (35)	1801 (43)		
Female	2247 (58)	305 (65)	1942 (57)		
Race, *n* (%)				*χ* ^2^ = 7.62	0.022
Non-Hispanic White	1836 (66)	192 (57)	1644 (67)		
Non-Hispanic Black	1022 (13)	115 (17)	907 (13)		
Others	1374 (21)	182 (26)	1192 (20)		
Education, *n* (%)				*χ* ^2^ = 15.61	< 0.001
Below high school	1292 (22)	202 (31)	1090 (20)		
High school	1046 (26)	113 (27)	933 (27)		
Above high school	1894 (52)	174 (42)	1720 (53)		
Marriage, *n* (%)				*χ* ^2^ = 16.26	< 0.001
Married	2067 (51)	185 (41)	1882 (52)		
Never married	499 (13)	71 (13)	428 (13)		
Others	1666 (36)	233 (46)	1433 (35)		
PIR, *n* (%)				*χ* ^2^ = 65.15	< 0.001
< 1.0	940 (17)	182 (32)	758 (15)		
≥ 1.0	2911 (75)	271 (61)	2640 (77)		
Unknown	381 (8)	36 (7)	345 (8)		
Smoking, *n* (%)				*χ* ^2^ = 8.61	0.003
Yes	2107 (50)	284 (59)	1823 (49)		
No	2125 (50)	205 (41)	1920 (51)		
Drinking, *n* (%)				*χ* ^2^ = 0.35	0.557
Yes	2738 (68)	318 (70)	2420 (68)		
No	1494 (32)	171 (30)	1323 (33)		
Physical activity, *n* (%)				*χ* ^2^ = 8.62	0.013
< 450 met⁣^∗^minutes/week	473 (12)	46 (12)	427 (12)		
≥ 450 met⁣^∗^minutes/week	2090 (53)	207 (43)	1883 (54)		
Unknown	1669 (35)	236 (44)	1433 (34)		
Sleep disorder, *n* (%)				*χ* ^2^ = 219.96	< 0.001
Yes	1323 (34)	296 (68)	1027 (30)		
No	2909 (66)	193 (32)	2716 (70)		
Thyroid disease, *n* (%)				*χ* ^2^ = 4.21	0.040
Yes	645 (18)	93 (22)	552 (17)		
No	3587 (82)	396 (78)	3191 (83)		
Diabetes, *n* (%)				*χ* ^2^ = 23.37	< 0.001
Yes	1798 (37)	263 (49)	1535 (35)		
No	2434 (63)	226 (51)	2208 (65)		
Hypertension, *n* (%)				*χ* ^2^ = 7.44	0.006
Yes	3130 (69)	379 (78)	2751 (68)		
No	1102 (31)	110 (22)	992 (32)		
Dyslipidemia, *n* (%)				*χ* ^2^ = 12.93	< 0.001
Yes	3424 (79)	409 (86)	3015 (79)		
No	808 (21)	80 (14)	728 (21)		
CVD, *n* (%)				*χ* ^2^ = 13.23	< 0.001
Yes	1860 (39)	252 (50)	1608 (37)		
No	2372 (61)	237 (50)	2135 (63)		
Dialysis, *n* (%)				*χ* ^2^ = 19.35	< 0.001
Yes	82 (1)	16 (2)	66 (1)		
No	434 (9)	75 (14)	359 (8)		
Unknown	3716 (90)	398 (84)	3318 (91)		
ACEI, *n* (%)				*χ* ^2^ = 1.88	0.170
Yes	1115 (24)	135 (28)	980 (24)		
No	3117 (76)	354 (72)	2763 (77)		
ARB, *n* (%)				*χ* ^2^ = 0.62	0.431
Yes	4 (1)	2 (1)	2 (1)		
No	4228 (99)	487 (99)	3741 (99)		
Glucocorticoids, *n* (%)				*χ* ^2^ = 3.47	0.062
Yes	143 (3)	29 (5)	114 (3)		
No	4089 (97)	460 (95)	3629 (97)		
Immunosuppressant, *n* (%)				*χ* ^2^ = 0.86	0.354
Yes	34 (1)	3 (1)	31 (1)		
No	4198 (99)	486 (99)	3712 (99)		
Nephrotoxic drugs, *n* (%)				*χ* ^2^ = 10.38	0.001
Yes	299 (7)	58 (10)	241 (6)		
No	3933 (93)	431 (90)	3502 (94)		
Psychotherapeutic agents, *n* (%)				*χ* ^2^ = 122.91	< 0.001
Yes	639 (18)	179 (43)	460 (15)		
No	3593 (82)	310 (57)	3283 (85)		
BMI (kg/m^2^), mean (SE)	30.42 (0.20)	32.67 (0.62)	30.15 (0.23)	*t* = −3.58	< 0.001
WBC (1000 cells/*μ*L), mean (SE)	7.69 (0.06)	7.96 (0.13)	7.66 (0.06)	*t* = −2.23	0.028
Neutrophil (%), mean (SE)	60.58 (0.22)	60.98 (0.64)	60.54 (0.22)	*t* = −0.69	0.495
Lymphocyte (%), mean (SE)	27.86 (0.19)	27.58 (0.61)	27.90 (0.20)	*t* = 0.50	0.621
Albumin (g/L), mean (SE)	41.66 (0.10)	41.05 (0.30)	41.73 (0.10)	*t* = 2.27	0.025
Hemoglobin (g/dL), mean (SE)	13.80 (0.05)	13.73 (0.11)	13.81 (0.05)	*t* = 0.67	0.503

*Note:χ* ^2^: chi-square tests.

Abbreviations: ACEI: angiotensin-converting enzyme inhibitor, and ARB: angiotensin-receptor blocker, BMI: body mass index, CKD: chronic kidney disease, CVD: cardiovascular disease, DII: dietary inflammation index, PIR: poverty income ratio, SE: standard error, *t*: *t*-tests, WBC: white blood cell.

**Table 2:** Association of DII score with depression symptoms among CKD patients.

**Variables**	**Model 1**		**Model 2**		**Model 3**	
	**OR (95% CI)**	**p**	**OR (95% CI)**	**p**	**OR (95% CI)**	**p**
DII score	1.21 (1.10–1.34)	< 0.001	1.18 (1.06–1.32)	0.003	1.15 (1.03–1.29)	0.015
DII score						
< 0.65	Ref		Ref		Ref	
0.65–2.14	1.34 (0.88–2.05)	0.174	1.28 (0.83–1.97)	0.264	1.14 (0.72–1.81)	0.565
≥ 2.14	2.03 (1.36–3.03)	< 0.001	1.85 (1.21–2.84)	0.005	1.67 (1.06–2.65)	0.028
*p* for trend	1.44 (1.19–1.75)	< 0.001	1.38 (1.12–1.70)	0.003	1.31 (1.05–1.65)	0.018

*Note:* Model 1: crude model. Model 2: adjusting age, gender, and race. Model 3: adjusting age, gender, race, smoking, sleep disorders, cardiovascular disease, and psychotherapeutic agents.

Abbreviations: CI: confidence interval; DII: dietary inflammation index; OR: odds ratio; Ref: reference.

**Table 3:** Associations of DII with depression symptoms in subgroups of age, gender, smoking, and sleep disorder.

**Variables**	**Adjusted model**						
	**OR (95% CI)**	**p**	**FDR ** **p**	**OR (95% CI)**	**p**	**FDR ** **p**	**p** ** for interaction**
Subgroup I: Age	< 60 years old (*n* = 1657)			≥ 60 years old (*n* = 2575)			0.662
DII score	1.16 (0.98–1.38)	0.088	0.176	1.13 (0.99–1.28)	0.069	0.176	
DII score							
< 0.65	Ref			Ref			
0.65–2.14	1.01 (0.47–2.20)	0.972	0.972	1.27 (0.83–1.94)	0.269	0.323	
≥ 2.14	1.49 (0.74–3.01)	0.258	0.323	1.80 (1.16–2.79)	0.009	0.054	
Subgroup II: Gender	Male (*n* = 1985)			Female (*n* = 2247)			0.450
DII score	1.22 (1.05–1.41)	0.009	0.039	1.12 (0.95–1.31)	0.176	0.264	
DII score							
< 0.65	Ref			Ref			
0.65–2.14	1.20 (0.70–2.07)	0.511	0.613	1.10 (0.58–2.08)	0.774	0.774	
≥ 2.14	2.05 (1.16–3.59)	0.013	0.039	1.50 (0.83–2.70)	0.174	0.264	
Subgroup III: Smoking	No (*n* = 2125)			Yes (*n* = 2107)			0.882
DII score	1.15 (0.97–1.36)	0.109	0.218	1.15 (1.00–1.33)	0.055	0.165	
DII score							
< 0.65	Ref			Ref			
0.65–2.14	1.36 (0.60–3.07)	0.451	0.541	0.98 (0.65–1.49)	0.941	0.941	
≥ 2.14	1.58 (0.75–3.31)	0.222	0.333	1.70 (1.06–2.75)	0.029	0.165	
Subgroup IV: Sleep disorders	No (*n* = 2909)			Yes (*n* = 1323)			0.514
DII score	1.19 (1.01–1.42)	0.043	0.135	1.13 (0.97–1.31)	0.116	0.205	
DII score							
< 0.65	Ref			Ref			
0.65–2.14	1.47 (0.88–2.45)	0.140	0.205	0.99 (0.51–1.93)	0.988	0.988	
≥ 2.14	1.81 (1.01–3.23)	0.045	0.135	1.57 (0.82–2.99)	0.171	0.205	
Subgroup V: Psychotherapeutic agents	No (*n* = 3593)			Yes (*n* = 639)			0.122
DII score	1.07 (0.93–1.23)	0.311	0.486	1.29 (1.08–1.55)	0.006	0.018	
DII score							
< 0.65	Ref			Ref			
0.65–2.14	1.22 (0.70–2.12)	0.475	0.570	1.04 (0.63–1.72)	0.865	0.865	
≥ 2.14	1.33 (0.75–2.35)	0.324	0.486	2.40 (1.33–4.33)	0.004	0.018	

*Note:* Subgroup I: adjusting gender, race, smoking, sleep disorders, cardiovascular disease, and psychotherapeutic agents. Subgroup II: adjusting age, race, smoking, sleep disorders, cardiovascular disease, and psychotherapeutic agents. Subgroup III: adjusting age, gender, race, sleep disorders, cardiovascular disease, and psychotherapeutic agents. Subgroup IV: adjusting age, gender, race, smoking, cardiovascular disease, and psychotherapeutic agents. Subgroup V: adjusting age, gender, race, smoking, cardiovascular disease, and sleep disorder.

Abbreviations: CI: confidence interval; DII: dietary inflammation index; OR: odds ratio; Ref: reference.

## Data Availability

The datasets generated and/or analyzed during the current study are available in the NHANES database, https://wwwn.cdc.gov/nchs/nhanes/.

## Nomenclature

CKD: chronic kidney disease
DII: dietary inflammatory index
NHANES: Nation Health and Nutrition Examination Survey
NCHS: National Center for Health Statistics
eGFR: estimated glomerular filtration rate
ACR: albumin–Cr ratio
PHQ-9: Patient Health Questionnaire-9
BMI: body mass index
CVD: cardiovascular disease
SE: standard errors
ORs: odds ratios
CIs: confidence intervals
RCS: restricted cubic spline

## Supporting Information

Supporting Information 1 Table S1: The screening of potential covariates.

Supporting Information 2 Table S2: The number and percentage of missing values.

Supporting Information 3 Table S3: Sensitivity analysis of the data before and after manipulation of the missing values.

Supporting Information 4 Table S4: The proportion of patients with depression in different subgroups.
