# Supplementary material for: Association Between Dietary Inflammatory Index and Depression Symptoms in Chronic Kidney Disease
Source: Behav Neurol. 2025 Mar 7;2025:9253956. doi: 10.1155/bn/9253956 (PMC11991767; doi:10.1155/bn/9253956)
Supplement: Supporting Information 1 — Table S1: The screening of potential covariates. [file 9253956.f1.docx]

Table S1 The screening of potential covariates

| Variables | OR (95%CI) | *P* |
| --- | --- | --- |
| Age |  |  |
| <60 | Ref |  |
| ≥60 | 0.68 (0.52-0.90) | 0.007 |
| Gender |  |  |
| Male | Ref |  |
| Female | 1.37 (1.04-1.81) | 0.024 |
| Race |  |  |
| Non-Hispanic White | Ref |  |
| Non-Hispanic Black | 1.49 (1.12-1.98) | 0.007 |
| Others | 1.50 (0.98-2.29) | 0.060 |
| Education |  |  |
| Below high school | Ref |  |
| High school | 0.65 (0.44-0.96) | 0.029 |
| Above high school | 0.52 (0.38-0.72) | <0.001 |
| Marriage |  |  |
| Married | Ref |  |
| Never married | 1.22 (0.82-1.80) | 0.317 |
| Others | 1.71 (1.28-2.30) | <0.001 |
| PIR |  |  |
| <1.0 | Ref |  |
| ≥1.0 | 0.37 (0.30-0.47) | <0.001 |
| Unknown | 0.42 (0.26-0.69) | <0.001 |
| Smoke |  |  |
| No | Ref |  |
| Yes | 1.50 (1.14-1.97) | 0.004 |
| Drink |  |  |
| No | Ref |  |
| Yes | 1.09 (0.82-1.44) | 0.558 |
| Physical activity |  |  |
| <450 met*minutes/week | Ref |  |
| ≥450 met*minutes/week | 0.75 (0.41-1.39) | 0.360 |
| Unknown | 1.24 (0.67-2.30) | 0.485 |
| Sleep disorder |  |  |
| No | Ref |  |
| Yes | 5.04 (3.93-6.44) | <0.001 |
| Thyroid disease |  |  |
| No | Ref |  |
| Yes | 1.34 (1.01-1.77) | 0.043 |
| Diabetes |  |  |
| No | Ref |  |
| Yes | 1.80 (1.41-2.29) | <0.001 |
| Hypertension |  |  |
| No | Ref |  |
| Yes | 1.67 (1.15-2.42) | 0.008 |
| Dyslipidemia |  |  |
| No | Ref |  |
| Yes | 1.65 (1.25-2.19) | <0.001 |
| CVD |  |  |
| No | Ref |  |
| Yes | 1.69 (1.27-2.25) | <0.001 |
| BMI | 1.04 (1.02-1.06) | <0.001 |
| WBC | 1.03 (1.01-1.06) | 0.022 |
| Neutrophil | 1.00 (0.99-1.02) | 0.498 |
| Lymphocyte | 1.00 (0.98-1.01) | 0.624 |
| Albumin | 0.95 (0.91-0.99) | 0.020 |
| Hemoglobin | 0.97 (0.90-1.05) | 0.503 |
| Dialysis |  |  |
| No | Ref |  |
| Yes | 1.06 (0.55-2.05) | 0.856 |
| Unknown | 0.53 (0.38-0.76) | <0.001 |
| ACEI |  |  |
| No | Ref |  |
| Yes | 1.25 (0.90-1.74) | 0.172 |
| ARB |  |  |
| No | Ref |  |
| Yes | 2.22 (0.28-17.52) | 0.445 |
| Glucocorticoids |  |  |
| No | Ref |  |
| Yes | 1.61 (0.96-2.68) | 0.069 |
| Immunosuppressor |  |  |
| No | Ref |  |
| Yes | 0.51 (0.12-2.18) | 0.363 |
| Nephrotoxic drugs |  |  |
| No | Ref |  |
| Yes | 1.76 (1.24-2.51) | 0.002 |
| Psychotherapeutic agents |  |  |
| No | Ref |  |
| Yes | 4.15 (3.18-5.43) | <0.001 |

OR: odds ratio; CI: confidence interval; Ref: reference
