# Supplementary material for: Association Between Dietary Inflammatory Index and Depression Symptoms in Chronic Kidney Disease
Source: Behav Neurol. 2025 Mar 7;2025:9253956. doi: 10.1155/bn/9253956 (PMC11991767; doi:10.1155/bn/9253956)
Supplement: Supporting Information 2 — Table S2: The number and percentage of missing values. [file 9253956.f2.docx]

Table S2 The number and percentage of missing values

| Variables | n(%) |
| --- | --- |
| Education | 125(2.95%) |
| Marriage | 121(2.86%) |
| Smoke | 72(1.7%) |
| Drink | 139(3.28%) |
| WBC | 148(3.5%) |
| Neutrophil | 159(3.76%) |
| Lymphocyte | 159(3.76%) |
| Albumin | 210(4.96%) |
| Hemoglobin | 148(3.5%) |
