# Supplementary material for: Association Between Dietary Inflammatory Index and Depression Symptoms in Chronic Kidney Disease
Source: Behav Neurol. 2025 Mar 7;2025:9253956. doi: 10.1155/bn/9253956 (PMC11991767; doi:10.1155/bn/9253956)
Supplement: Supporting Information 3 — Table S3: Sensitivity analysis of the data before and after manipulation of the missing values. [file 9253956.f3.docx]

Table S3 Sensitivity analysis of the data before and after manipulation of the missing values

| Variables | Before | After | Statistics | *P* |
| --- | --- | --- | --- | --- |
| Education, n(%) |  |  | χ^2^=2.49 | 0.288 |
| Below high school | 1260 (21.42) | 1292 (21.31) |  |  |
| High school | 1013 (26.73) | 1046 (26.62) |  |  |
| College and above | 1834 (51.85) | 1894 (52.07) |  |  |
| Marriage, n(%) |  |  | χ^2^=2.94 | 0.229 |
| Married | 1999 (51.81) | 2067 (51.95) |  |  |
| Never married | 481 (11.96) | 499 (12.07) |  |  |
| Others | 1631 (36.23) | 1666 (35.99) |  |  |
| Smoke, n(%) |  |  | χ^2^=0.12 | 0.731 |
| No | 2090 (50.08) | 2125 (50.13) |  |  |
| Yes | 2070 (49.92) | 2107 (49.87) |  |  |
| Drink, n(%) |  |  | χ^2^=0.86 | 0.353 |
| No | 1437 (31.88) | 1494 (32.00) |  |  |
| Yes | 2656 (68.12) | 2738 (68.00) |  |  |
| WBC, 1000 cells/uL, Mean (S.E) | 7.64 (0.06) | 7.64 (0.05) | t=-0.09 | 0.931 |
| Neutrophil, %, Mean (S.E) | 60.50 (0.22) | 60.52 (0.21) | t=-0.91 | 0.366 |
| Lymphocyte, %, Mean (S.E) | 27.89 (0.19) | 27.87 (0.19) | t=0.51 | 0.614 |
| Albumin, g/L, Mean (S.E) | 41.56 (0.10) | 41.58 (0.10) | t=-1.57 | 0.119 |
| Hemoglobin, g/dL, Mean (S.E) | 13.80 (0.05) | 13.81 (0.05) | t=-1.40 | 0.164 |

t, t tests; χ^2^, chi-square tests; S.E: standard error
