# Supplementary material for: Association Between Dietary Inflammatory Index and Depression Symptoms in Chronic Kidney Disease
Source: Behav Neurol. 2025 Mar 7;2025:9253956. doi: 10.1155/bn/9253956 (PMC11991767; doi:10.1155/bn/9253956)
Supplement: Supporting Information 4 — Table S4: The proportion of patients with depression in different subgroups. [file 9253956.f4.docx]

Table S4 Proportion of patients with depression in different subgroups

| Variables | Depression | Non-depression |
| --- | --- | --- |
| Age |  |  |
| <60 | 12.28 | 87.72 |
| ≥60 | 8.7 | 91.30 |
| Gender |  |  |
| Male | 8.71 | 91.29 |
| Female | 11.6 | 88.4 |
| Smoke |  |  |
| No | 8.5 | 91.5 |
| Yes | 12.3 | 87.7 |
| Sleep disorder |  |  |
| No | 5 | 95 |
| Yes | 21 | 79 |
| Psychotherapeutic agents |  |  |
| No | 7.24 | 92.76 |
| Yes | 24.5 | 75.5 |
